# Ecological Momentary Assessment of Adolescent Problems, Coping Efficacy, and Mood States Using a Mobile Phone App: An Exploratory Study

**DOI:** 10.2196/mental.6361

**Published:** 2016-11-29

**Authors:** Rachel Kenny, Barbara Dooley, Amanda Fitzgerald

**Affiliations:** ^1^ Youth Mental Health Lab School of Psychology University College Dublin Dublin Ireland

**Keywords:** adolescent, affect, ecological momentary assessment, mobile apps

## Abstract

**Background:**

Mobile technologies have the potential to be used as innovative tools for conducting research on the mental health and well-being of young people. In particular, they have utility for carrying out ecological momentary assessment (EMA) research by capturing data from participants in real time as they go about their daily lives.

**Objective:**

The aim of this study was to explore the utility of a mobile phone app as a means of collecting EMA data pertaining to mood, problems, and coping efficacy in a school-based sample of Irish young people.

**Methods:**

The study included a total of 208 participants who were aged 15-18 years, 64% female (113/208), recruited from second-level schools in Ireland, and who downloaded the CopeSmart mobile phone app as part of a randomized controlled trial. On the app, participants initially responded to 5 single-item measures of key protective factors in youth mental health (formal help-seeking, informal help-seeking, sleep, exercise, and sense of belonging). They were then encouraged to use the app daily to input data relating to mood states (happiness, sadness, anger, stress, and worry), daily problems, and coping self-efficacy. The app automatically collected data pertaining to user engagement over the course of the 28-day intervention period. Students also completed pen and paper questionnaires containing standardized measures of emotional distress (Depression, Anxiety, and Stress Scale; DASS-21), well-being (World Health Organization Well-Being Index; WHO-5), and coping (Coping Strategies Inventory; CSI).

**Results:**

On average the participants completed 18% (5/28) of daily ratings, and engagement levels did not differ across gender, age, school, socioeconomic status, ethnicity, or nationality. On a scale of 1 to 10, happiness was consistently the highest rated mood state (overall mean 6.56), and anger was consistently the lowest (overall mean 2.11). Pearson correlations revealed that average daily ratings of emotional states were associated with standardized measures of emotional distress (*r*_happiness_=–.45, *r*_sadness_=.51, *r*_anger_=.32, *r*_stress_=.41, *r*_worry_=.48) and well-being (*r*_happiness_=.39, *r*_sadness_ =–.43, *r*_anger_=–.27, *r*_stress_=–.35, *r*_worry_=–.33)_._ Inferential statistics indicated that single-item indicators of key protective factors were related to emotional distress, well-being, and average daily mood states, as measured by EMA ratings. Hierarchical regressions revealed that greater daily problems were associated with more negative daily mood ratings (all at the *P*<.001 level); however, when coping efficacy was taken into account, the relationship between problems and happiness, sadness, and anger became negligible.

**Conclusions:**

While engagement with the app was low, overall the EMA data collected in this exploratory study appeared valid and provided useful insights into the relationships between daily problems, coping efficacy, and mood states. Future research should explore ways to increase engagement with EMA mobile phone apps in adolescent populations to maximize the amount of data captured by these tools.

**Trial Registration:**

Clinicaltrials.gov NCT02265978; http://clinicaltrials.gov/ct2/show/NCT02265978 (Archived by WebCite at http://www.webcitation.org/6mMeYqseA).

## Introduction

Mobile technologies have the potential to be used as innovative tools for conducting research on the mental health and well-being of young people. In particular, they have utility for carrying out ecological momentary assessment (EMA) [1] or experience sampling methods [2]. These methodological terms (hereafter denoted as EMA) refer to the process of capturing data from participants in real time as they go about their daily lives [1-3] and are especially useful for exploring dynamic constructs such as symptoms of psychopathology and affective states, which tend to fluctuate over time [2,4].

A key advantage of EMA is that it addresses the limitations presented by retrospective measures of behaviors and experiences. Autobiographical memory is considered to be a representation of experience, largely reconstructed through the use of heuristic strategies, which can cause recall to become biased [1]. For example, the availability heuristic postulates that individuals judge events to be more frequent if they are easily retrievable from memory [5]. This heuristic produces accurate estimates of frequency in cases where an event is easily retrievable due to familiarity with that event. However, biases can occur when events are easily retrievable for other reasons (eg, because they occurred recently or because they were emotionally salient), which may result in overestimation of their frequency [1]. Another limitation of autobiographical memory is the potential for memories of a situation to be unconsciously distorted based on preexisting expectancies, thoughts, and beliefs about that situation [1,6]. In a research context, this can affect the validity of retrospective measures of behaviors and affective states, particularly where such measures have not been well validated in the population under study. For example, research suggests that participants tend to overestimate intensity and duration of symptoms in recall-based measures [1]. EMA overcomes these issues by assessing participants’ states in real time as they are occurring, thus providing more reliable and ecologically valid measurements.

EMA is not new to social research [2]. In past studies, young people have been given pencil and paper diaries to complete real-time measures of behaviors and moods [7-9]. However, these methods raised concerns around compliance, whereby participants would not complete measures at the correct time and would “backfill” them on a later occasion, or, in some cases, would fill in measures ahead of time rendering the data invalid [10]. Advances in mobile technologies have resulted in a means of addressing this issue due to the increasing availability of electronic methods of data collection, such as mobile phones, which provide time and date stamps for data entered by participants.

Indeed, mobile phones have the potential to be a particularly valuable means of collecting EMA data. These technologies are now almost ubiquitous, with ownership of smartphones (ie, mobile phones that can connect to data networks such as the Internet) almost doubling among American adults between 2011 and 2015 [11], and adolescent smartphone ownership is estimated at between 75% and 86% in the developed world [11-13]. Research also suggests that smartphone ownership is not restricted by socioeconomic status [14]. Thus, they represent a widely available and highly accessible medium for capturing data about diverse populations. Furthermore, as individuals are used to carrying their phones around with them, the likelihood of missing entries due to participants forgetting to bring an additional research device with them is reduced. Mobile phones also have value as being potentially very cost-effective data collection tools, as they are highly scalable. Although initial investment may be required to develop the data collection software platform, it is much simpler to subsequently customize that platform to capture different types of data and answer different research questions [15]. Furthermore, researchers no longer need to purchase mobile devices for participants, as software applications—more commonly known as mobile phone apps [16]—for collecting data can be downloaded directly onto participants’ personal mobile phone devices. Thus, collecting data from large numbers of participants is unlikely to entail additional costs, making large-scale projects a more accessible and feasible option for researchers.

Recent years have seen frequent use of mobile-based EMA methods in studies with young people, particularly in relation to health behaviors such as medication adherence [17], smoking [18], and eating behaviors [19]. EMA methods have also been increasingly used to study emotional states in adolescents with clinically diagnosed mental health problems such as mood and anxiety disorders [3,20-22]. However, fewer studies have been conducted on the utility of electronic EMA methods to capture data pertaining to mood (ie, the experience of a current emotion such as happiness, sadness, anger, and so forth) and coping efficacy in a general, nonclinical adolescent population.

A pilot study by Abbot et al explored the feasibility of using EMA with a school-based sample of 40 Australian adolescents to capture data pertaining to their contextual environment, behaviors, and mood states [2]. They reported that not only was this method of capturing data feasible, but the young people involved in the study found the use of a mobile phone as a means of data collection to be particularly engaging. However, they did not report details of engagement or attrition. Furthermore, due to the small sample size, their analyses were only descriptive, and they did not examine the validity of the EMA data captured.

Another small-scale feasibility study was conducted by Garcia et al. They used EMA to collect data pertaining to daily activities, behaviors, and attitudes among 24 female Latina adolescents in the United States via mobile phones [23]. They tested 2 methods of EMA: one where participants were required to respond as quickly as possible to questions sent via text message (signal-based assessment) and one where participants were instructed to respond to text message–based questions whenever they wanted, but to provide unprompted open-ended texts describing how they felt during events that occurred in their daily lives (event-based assessment). They ran numerous rounds of data collection, with participants experiencing both methods at some point during the study. They found that compliance rates varied across both methods, with signal-based sampling inducing a higher compliance rate (average percentage of texts responded to was 79%) than event-based sampling (average percentage of texts responded to was 54%). However, their analyses were limited to examining compliance rates, and they did not present details of the actual EMA responses captured. Furthermore, the homogeneity of the sample (all Latina females) limits the generalizability of their findings.

Another example of a mobile phone-based method of mood-related EMA is Kauer et al’s intervention, Mobiletype [24]. This was a targeted mobile phone app, where Australian adolescents (N=68) with elevated levels of depression monitored their mood, stress, coping strategies, activities, eating, sleeping, exercise, and substance use. They found that engagement was moderate with participants completing EMA ratings 3-4 times per day on an average of 17-18 days within the 4-week intervention period, demonstrating the feasibility of this tool in a sample of adolescents experiencing depressive symptoms. However, the focus of their study was on evaluating the effectiveness of the app as an intervention. Thus, they did not report details of the EMA responses or examine their validity in comparison to standardized measures.

Overall, EMA has potential to provide an innovative, ecologically valid means of capturing detailed data about young people’s real-world experiences in their natural environments. Given the elevated prevalence of emotional problems experienced during adolescence [25,26], this is a particularly salient time to examine individuals’ daily experience of mood, problems, and coping efficacy. In light of the advantages of mobile phones as highly accessible and cost-effective tools for EMA research, it is important to explore their potential feasibility for studying such constructs in young people.

However, while small-scale studies have suggested the feasibility of mobile phone–based EMA pertaining to mental health in small adolescent samples [2,23,24], actual analyses of the EMA data captured in these studies has been limited or nonexistent and the validity of this data has not been explored. There has been increased recognition in recent years of the importance of promoting well-being in the general population of young people, in order to maximize beneficial outcomes, rather than just focusing on treating symptoms in young people already experiencing clinical levels of distress. Thus, it is important that we study the use of EMA in community-based adolescent samples. This is important for establishing the validity of such data and determining what it can tell us about young people’s daily experience of mood, problems, and coping efficacy, which will help inform the design and delivery of resources to support young people’s day-to-day well-being.

The aim of this study was to address the gap in the literature by exploring the utility of a mobile phone app as a means of collecting EMA data pertaining to mood, problems, and coping efficacy in a school-based sample of young people. The app used to collect data in our study was CopeSmart *.* This app was designed as a mental health intervention promoting emotional self-monitoring and positive coping strategies in adolescents [27]. As part of the intervention, users were encouraged to engage in self-monitoring by inputting EMA data to the app pertaining to their daily experience of problems, coping efficacy, and mood states. A more detailed description of the full app content is presented in Kenny, Dooley, and Fitzgerald [27].

As this study was exploratory, specific hypotheses were not generated. However, the researchers were interested in answering the following 4 general questions:

- First, the researchers were interested in exploring: (1) To what extent do participants engage with the EMA component of the app?
- Second, the researchers aimed to explore the validity of the data by testing: (2) Are EMA responses correlated with standardized measures of mental health and coping?
- As EMAs are completed during participants’ daily lives, it is intuitive that measures used should be brief in order to maximize participants’ responses. Thus, the researchers were interested in evaluating the use of single-item measures of key protective factors in youth mental health (described below) by assessing: (3) Are brief single-item measures of key protective factors related to adolescents’ daily mood states?
- Finally, research suggests that experiencing daily stressors, such as negative events and problems, is associated with lower positive mood and higher negative mood during adolescence [28,29]. Studies also indicate that coping effectively with these stressors is associated with more positive mood outcomes in young people [30-32]. The EMA data collected by the app in this study offered a highly ecologically valid means of generating insight into the relationships between these variables in an adolescent sample. Thus, the researchers were interested in exploring (4) What is the relationship between daily problems, coping efficacy, and mood states in young people, as measured by EMA data?

## Methods

### Participants

The study included a total of 208 participants, 64% females (133/208), aged 15-18 years (mean 15.98, SD 0.70), recruited from 10 schools in the Republic of Ireland, and for whom mobile phone app data were captured as part of an effectiveness evaluation of the CopeSmart app intervention to be presented in a separate paper (Clinicaltrials.gov NCT02265978). In terms of nationality, 95% of the sample (197/208) were Irish and the remaining 5% were foreign nationals. In terms of ethnicity, 97% (202/208) identified as white, 0.5% (1/208) identified as Asian, and 1.5% (3/208) identified as black or “other.” In terms of socioeconomic status, 37% (77/208) attended schools that were considered to be socially or economically disadvantaged based on nationally established governmental criteria [33].

### Measures

#### EMA Data

**Figure 1:**
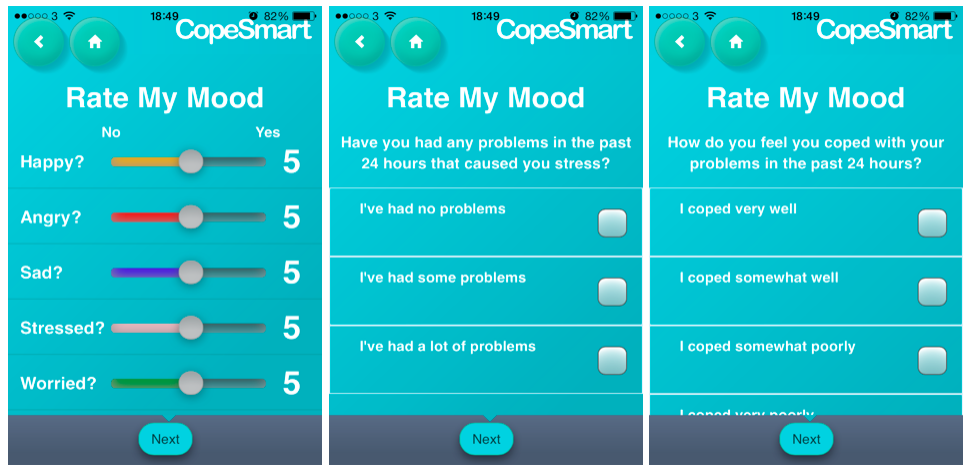
Sample screenshots from the ecological monetary assessment (EMA) component of the CopeSmart app.

The CopeSmart app contained a self-monitoring component where users recorded information about their mood states, problems, and coping efficacy (See Figure 1 for sample screenshots). The 3 types of self-report EMA data collected were as follows:

- Mood states: Participants recorded how happy, angry, sad, stressed, or worried they felt using a sliding scale ranging from 1 to 10. When users initially navigated to this page of the app, the sliders were by default centered in the middle of the scale and it was up to the users to slide them up or down to select the value of their mood rating.
- Experience of problems: Participants recorded their experience of problems in the last 24 hours (response options: “I’ve had no problems,” “I’ve had some problems,” “I’ve had a lot of problems”).
- Coping efficacy: Participants reported how well they felt they coped with these problems (response options: “I coped very well,” “I coped somewhat well,” “I coped somewhat poorly,” “I coped very poorly”).

Data relating to user’s engagement with the app (ie, how frequently they logged onto the app) were also captured. All EMA data were uploaded to a back-end server when the device came into contact with a wireless Internet connection.

#### Standardized Psychometric Scales

##### The Depression, Anxiety, and Stress Scale (DASS-21)—Short Version

The DASS-21 [34] is a 21 item self-report measure that assesses overall levels of psychological distress. It comprises 3 subscales representing 3 negative emotional states: depression, anxiety, and stress. Responses are made on a 4-point Likert scale based on the participants’ experiences of the past week, ranging from “Did Not Apply To Me At All” to “Applied To Me Most Of The Time.” Higher scores indicate higher levels of distress. The DASS-21 has shown convergent validity with other measures of negative affect [35], and it has been found to have a high level of internal reliability as an overall measure of distress (alpha=.93-.94) in adolescent samples [36,37] (this study, alpha=.92).

##### World Health Organization Well-Being Index (WHO-5)

The WHO-5 [38] is a brief 5-item self-report instrument developed from the World Health Organization-Ten Well-Being Index [39,40]. It is a unidimensional measure of positive psychological well-being containing 5 positively worded items pertaining to general well-being. Participants rate the degree to which they have experienced each of these positive feelings in the last 2 weeks, on a 6-point scale ranging from “At No Time” to “All of the Time,” with higher scores indicating higher levels of well-being. The scale’s 1-factor structure has been confirmed, and satisfactory concurrent validity has been established with other mental health measures [41,42]. It has been shown to consistently display a high level of internal reliability (alpha=.82-.89) in adolescent samples [41,43] (this study, alpha=.85).

##### The Coping Strategies Inventory (CSI)—Short Form

The 32-item short version of the self-report CSI [44] was designed to assess coping skills in young people on multiple levels. Partially derived from the Folkman and Lazarus (1980) Ways of Coping Scale, the measure assesses both positive “engagement” coping strategies and negative “disengagement” coping strategies. Participants were asked to indicate how much they used these strategies to cope with problems on 5-point scale ranging from “Never” to “Very Often,” with higher scores indicating more frequent use of that coping strategy. The CSI has shown satisfactory internal consistency (alpha=.69-.94) [44,45] and good test-retest reliability (*r*=.67-.83) in young adults [44] and concurrent validity with other measures of coping [46] (this study, alpha_engagement_=.89, alpha_disengagement_=.86).

#### Well-Being Indicator Items

When participants initially downloaded the app, they were asked 5 single-item “well-being indicator” questions pertaining to known protective factors for mental health outcomes in young people. These were informal help-seeking, formal help-seeking, physical activity, sleep, and sense of connectedness.

##### Informal Help-Seeking

Informal help-seeking was assessed by asking participants “When you have problems do you usually talk about then with anyone?’’ to which they could respond either “yes” or “no.” Talking about problems has been linked to more positive mental health outcomes in young people [47-49], and this particular item has previously been shown to a key indicator of mental health status in a sample of Irish adolescents [25].

##### Formal Help-Seeking

Formal help-seeking is associated with lower levels of distress in young people [50,51], and in this study, it was assessed using an item adapted from Saunders et al [52] that has previously been used in an Irish adolescent samples [25]. This item is based on the idea of formal help-seeking as a 3-stage process that involves identifying oneself as having a serious problem, recognizing that professional help is required, and actively seeking help [51]. Participants were asked “Have you had any serious problems in the last month?” Response options were “I’ve had few or no problems,” “I’ve had some problems, but I did not feel I needed professional help,” “I’ve had some problems but I did not seek professional help although I thought I needed it,” and “I’ve had some problems and I did seek professional help.”

##### Physical Activity

Besides promoting physical well-being, engagement in physical activity has been continuously linked with more positive mental health outcomes among adolescents [53-55]. The World Health Organization’s [56] Global Recommendations on Physical Activity for Health advise that, for optimum physical and mental health, children and adolescents should engage in at least sixty minutes of moderate-to-vigorous physical activity daily. Engagement in physical activity in this study was assessed using a single-item measure, devised and validated in an adolescent population [57]. Participants were given the statement “Physical activity is any activity that increases your heart rate and makes you a little out of breath e.g. running, fast walking, cycling, dancing etc.” and asked “On a usual week, how many days are you physically active for a total of at least 60 minutes per day?”

##### Sleep

A recent systematic review by Shochat et al [58] identified insufficient sleep as having a strong bidirectional relationship with depression during adolescence, as well as being linked to anxiety, poor psychosocial function, and poor perceived mental health. The National Sleep Foundation [59] ascertains that adolescents need between 8 and 10 hours of sleep each night for optimal functioning. Thus, in this study, participants were asked “During the past month, how many hours of sleep did you get on average per night?”

##### Sense of Connectedness

Finally, feeling a sense of connectedness and belonging to the people around oneself is considered to be a fundamental psychological need [60] and is associated with lower levels of depressive symptoms [61,62] and higher levels of well-being among young people [60]. In this study, it was measured by asking participants “Please indicate how much the following statement applies to you: I feel a sense of connectedness and belonging to people around me.” Responses were made on a 4-point scale ranging from “None of the time” to “All of the time.”

### Procedure

Ethical approval for this study was granted by the Human Research Ethics Committee—Humanities in the authors’ university (reference number: HS-13-45-Kenny-Dooley). Data collection took place between October 2014 and May 2015. Presented here is an overview of the procedure relevant to this study; a more detailed description of the full sampling, recruitment, and randomization procedure for the trial will be presented in a separate paper.

Initially, principals and guidance counselors of second-level schools in the Republic of Ireland were contacted. The nature of the study and what would be involved if the school decided to participate was described to them. In schools that agreed to take part, potential participants were provided with information sheets and consent forms for themselves and their parents or guardians. Participation was voluntary, and monetary incentives to take part were not offered. Students who returned signed parental consent forms were eligible to take part and were required to sign assent forms prior to participation.

Schools were randomly assigned to either the intervention or control condition. Students in schools assigned to the intervention condition completed pen-and-paper questionnaires. These contained items pertaining to demographic information and the standardized psychometric measures detailed earlier. They were then given instructions for how to download and use the CopeSmart app and asked to try to engage with it once a day over the course of the following 4 weeks. Students in schools assigned to the control condition completed the same questionnaires but received no intervention, and are not included in the analyses for this study.

In order to link their questionnaire responses with their app data over time, students were required to generate a personalized 9-digit Anonymous Identification Code, designed to make sure that their information would remain anonymous to the researchers. They recorded this on their pen-and-paper questionnaire and input it to the app upon initial download.

By default the app was set to prompt participants to complete EMA mood ratings at 8 p.m. each evening. Users were provided with the option to subsequently change the time at which they received these notifications if they wished. Users were also free to access the other components of the app (such as viewing their mood rating history) at their leisure, even if they had not completed an EMA rating that day.

### Analyses

Responses from pen-and-paper questionnaires were input to SPSS version 20.0.0 (IBM). Mobile phone app data (ie, participants’ EMA ratings and details of their engagement levels with the app) were downloaded to Microsoft Excel and then transferred into SPSS. All analyses were run using SPSS. Basic descriptive and inferential statistics were run to explore participants’ engagement with the app and whether engagement levels differed across demographic variables. Pearson correlations were conducted to test the relationship between EMA ratings and standardized measures of mental health and coping efficacy. Various inferential analyses were used to examine whether mental health indicator variables were linked to mental health outcomes. Where multiple comparisons were conducted simultaneously, the rough false discovery rate correction was used to control for increased chance of Type 1 error occurring [63] using the formula [(n+1)/2n] x (.05), where n is the number of tests. In cases where post-hoc analyses were required for significant one-way analysis of variance (ANOVA), Scheffe post-hoc analyses were carried out if homogeneity of variance (HOV) was observed, and Dunnet C analyses were carried out if HOV was not observed. Correlations and regressions were used to explore relationships between problems, coping efficacy, and mood as measured by EMA.

## Results

### To What Extent Do Participants Engage With the EMA Component of the App?

Of the 208 participants who downloaded the app, no EMA data were recorded for 28% (58/208) of participants, a single EMA entry was recorded for 10% of participants (21/208), and repeated EMA data were present for 62% (129/208) of participants. In cases where participants completed EMA ratings more than once during the course of a given day, their mood ratings were averaged to provide a single score for each mood state for that particular 24-hour period. In cases where they had multiple responses to the problems and coping EMA items within 1 day, their last entry for that day was taken as the best representation of whether they had experienced problems in the last 24 hours and how well they felt that they had coped with them.

The number of days on which participants completed ratings ranged from 0 to 24, and engagement levels did not differ across gender, age, school socioeconomic status, ethnicity, or nationality. On average, among the 208 participants, users only completed ratings on 5 days (SD 5.44) within the 28-day intervention period, corresponding to an average engagement rate of 18% (calculated as 5 days, expressed as a percentage of 28 days). Looking to general app usage data indicated no substantial difference between the number of days on which participants accessed the app (mean 5, SD 5.52) and the number of days on which they completed EMA ratings, suggesting that participants likely completed EMA ratings every time they used the app. The number of participants who completed EMA ratings each day continually declined over the course of the 28 days; Multimedia Appendix 1 presents a chart depicting the number of participants who completed EMA ratings on each day of the intervention period. In terms of daily mood ratings, happiness was consistently the highest rated mood state (overall mean 6.56), and anger was consistently the lowest rated (overall mean 2.11). Generally sadness (overall mean 2.63) was rated lower than both worry (overall mean 3.45) and stress (overall mean 3.57), whereas worry and stress often overlapped with each other in terms of how highly they were rated throughout the intervention period. Multimedia Appendix 2 presents a chart depicting average EMA mood rating scores on each day of the intervention period.

### Are EMA Responses Correlated With Standardized Measures of Mental Health and Coping?

EMA ratings of participants’ daily problems ratings were coded 1 (“I’ve had no problems”), 2 (“I’ve had some problems”), or 3 (“I’ve had a lot of problems”). These daily ratings were summed to give an overall score, which was divided by the number of daily ratings the participant completed, in order to obtain an average daily problem score for each individual. Higher scores indicated greater daily experience of problems.

Similarly, participants’ EMA ratings of how well they felt they had coped with problems were coded 1 (“I coped very poorly”), 2 (“I coped somewhat poorly”), 3 (“I coped somewhat well”), or 4 (“I coped very well”). These ratings were summed to give an overall score and then divided by the number of days on which the participant completed a daily rating in order to give an average daily coping self-efficacy score for each individual. Higher scores indicated more effective coping.

For each of the 5 moods (happy, sad, angry, stressed, and worried), an average EMA mood score for each participant was computed by summing all of their ratings for that mood and dividing it by the number of days on which they completed ratings (descriptive statistics for these are presented in Table 1). As skewness and kurtosis values for all variables fell within an acceptable range [64], they were considered to approximate normality and parametric statistics were used in further analyses.

**Table 1:** Descriptive statistics for average ecological momentary assessment (EMA) variables.

Variable	Overall mean (SD)	Skewness	Kurtosis
Average problems	1.65 (0.45)	0.30	−0.26
Average coping efficacy	3.17 (0.63)	−0.79	0.94
Average sadness	2.74 (2.17)	0.81	0.63
Average happiness	6.36 (1.84)	−0.22	0.48
Average anger	2.40 (1.99)	1.15	1.86
Average stress	3.89 (2.46)	0.20	−0.72
Average worry	3.39 (2.31)	0.38	−0.44
			

Participants’ average daily experience of problems was positively correlated with negative coping strategies (as measured by the CSI; *r*=.31, *P*<.001), but was not correlated with positive coping strategies, as measured by the CSI. Participants’ perceived coping efficacy in relation to these problems was positively associated with positive coping strategies (*r*=.29, *P*<.001) and negatively associated with negative coping strategies (*r*=-.27, *P*<.001).

Average daily happiness was negatively correlated with emotional distress, as measured by the DASS-21 (*r*=–.45, *P*<.001), and positively correlated with well-being, as measured by the WHO-5 (*r*=.39, *P*<.001). Average daily negative emotions (sadness, anger, stress, and worry) were all significantly positively associated with distress (*r*_sadness_=.51, *P*<.001; *r*_anger_=.32, *P*<.001; *r*_stress_=.41, *P*<.001; *r*_worry_=.48, *P*<.001) and negatively correlated with well-being (*r*_sadness_=–.43, *P*<.001; *r*_anger_=–.27, *P*=.001; *r*_stress_=–.35, *P*<.001; *r*_worry_=–.33, *P*<.001).

### Are Brief Single-Item Measures of Key Protective Factors Related to Daily Mood States?

#### Informal Help-Seeking

About 55% (114/208) of participants reported that when they had problems they usually talked about them with someone. The remaining 45% (94/208) reported that they did not talk about their problems. A series of *t* tests revealed that those who reported that they talked about their problems displayed significantly lower levels of average daily anger *t*_109.27_=–2.26, *P*=.03 (mean_talk_=2.07, SD 1.97 vs mean_donotalk_ =2.83, SD 2.35) and significantly higher average daily happiness *t*_148_=2.48, *P*=.01 (mean_talk_=6.69, SD 1.60 vs mean_donotalk_=5.95, SD 2.04). There were no differences between the 2 groups in terms of daily sadness, stress, or worry.

#### Formal Help-Seeking

Overall, 49% of participants (101/208) had few or no problems, 36% (76/208) had some problems but did not feel they needed professional help, 10% (20/208) had some problems, felt they needed professional help, but did not seek it, and 5% (11/208) had problems and did seek professional help. Table 2 presents the results of One-way ANOVAs assessing whether participants who categorized themselves into different formal help-seeking categories differed in terms of daily mood states, as assessed by EMA.

Findings revealed that those who categorized themselves as having no problems reported less negative daily mood states than those who categorized themselves as having problems. Additionally, those who categorized themselves as having problems, but who did not feel they needed to seek help reported significantly lower daily sadness than those who categorized themselves as having problems and needing help. Among those who categorized themselves as needing help, no differences were evident between those who had sought and those who had not sought professional help.

**Table 2:** One-way analysis of variances (ANOVAs) examining differences between formal help-seeking groups in terms of average daily mood states.

Average EMA^a^ rating	Few or no problems, mean (SD)	Some problems, did not need help, mean (SD)	Some problems, needed help, did not seek it, mean (SD)	Some problems, sought professional help, mean (SD)	*F*	*P* ^b^	Post-hoc (Scheffe)
Happy	7.12 (1.64)	5.67 (1.64)	4.97 (1.87)	6.31 (1.59)	12.0^c^	<.001	1>2,3
Sad	1.78 (1.83)	3.36 (1.89)	5.03 (2.08)	3.29 (1.97)	16.98^c^	<.001	1<2,3; 2<3
Angry	1.85 (1.86)	2.94 (1.87)	2.94 (2.41)	3.18 (1.93)	4.25^d^	.007	1<2
Stressed	2.81 (2.34)	4.61 (2.17)	5.98 (1.76)	5.13 (1.57)	14.0^c^	<.001	1<2,3,4
Worried	2.35 (2.18)	4.13 (2.02)	5.07 (1.48)	5.03 (2.05)	14.15^d^	<.001	1<2,3,4

^a^EMA: ecological monetary assessment.

^b^Alpha set at .028 in line with rough false discovery rate.

^c^Significant at the *P*<.001 level.

^d^Significant at the *P*<.028 level.

**Figure 2:**
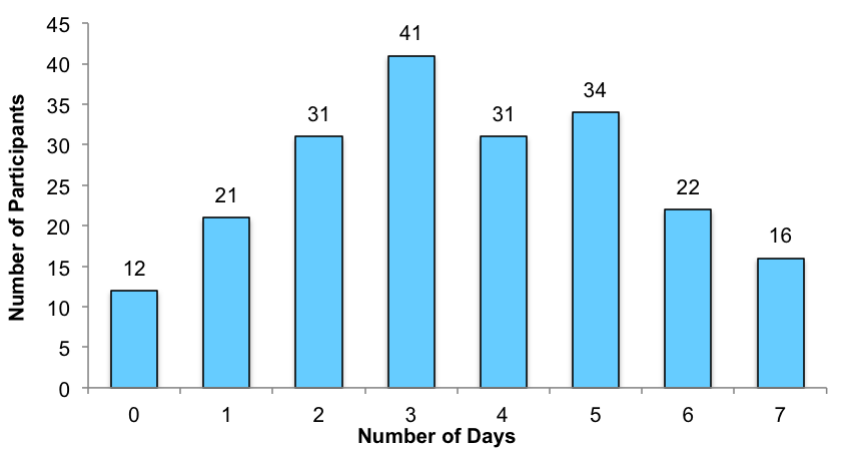
Days physically active in a typical week as reported by participants.

#### Physical Activity

Figure 2 illustrates the typical weekly activity levels reported by participants. Pearson correlations revealed no relationships between the average number of days on which participants were physically active and average EMA mood ratings.

#### Sleep

**Figure 3:**
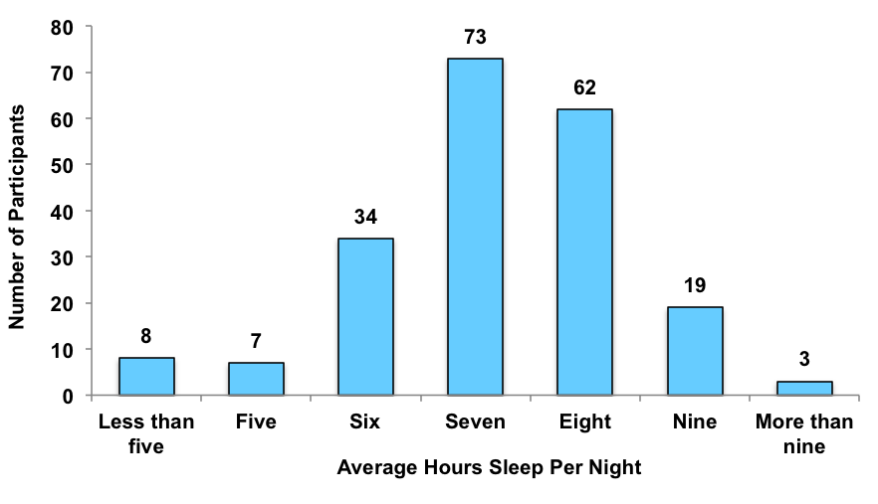
Participants’ average hours sleep per night.

Figure 3 illustrates participants’ average hours sleep per night over the course of the previous month (data were missing for 2 participants). Overall participants reported an average of 7.09 hours sleep (SD 1.60) per night. Independent *t*-tests (detailed in Table 3) were conducted examining differences between those who reported getting sufficient sleep (84/206) and those who did not (122/206). Sufficient sleep was defined as ≥8 hours in line with the National Sleep Foundation guidelines [59]. Findings revealed that adolescents who did not get sufficient sleep reported higher average daily sadness, anger, and worry and lower daily happiness.

#### Sense of Connectedness

Exactly half of participants (104/208) reported that they felt a sense of connectedness and belonging to those around them a lot or all of the time. The other half of participants reported that they felt this only some or none of the time. Table 3 presents the results of independent *t* tests examining differences between those who felt a sense of connectedness a lot or all of the time and those who felt this some or none of the time. Those who felt connectedness a lot or all of the time reported significant higher daily happiness and significantly lower daily sadness, anger, stress, and worry.

**Table 3:** *t* tests examining differences in average daily mood ratings across sleep groups and sense of connectedness groups.

Variable		Mean (SD)	Mean (SD)	*t*	*P* ^a^
**Sleep**		Insufficient sleep (<8 hours)	Sufficient sleep (≥8 hours)		
	Happy	5.91 (1.77)	6.92 (1.79)	–3.43^b^	.001
	Sad	3.22 (2.15)	2.16 (2.06)	3.06^b^	.003
	Angry	2.73 (2.07)	1.99 (1.83)	2.29^b^	.02
	Stressed	4.19 (2.22)	3.51 (2.70)	1.65	.10
	Worried	3.78 (2.31)	2.90 (2.24)	2.36^b^	.02
**Sense of connectedness**		Some or none of the time	A lot or all of the time		
	Happy	5.96 (1.83)	6.82 (1.75)	–2.92^b^	.004
	Sad	3.39 (2.27)	2.00 (1.79)	4.13^c^	<.001
	Angry	3.03 (2.17)	1.69 (1.49)	4.46^c^	<.001
	Stressed	4.48 (2.35)	3.21 (2.43)	3.24^b^	.001
	Worried	4.13 (2.45)	2.54 (1.82)	4.57^c^	<.001

^a^Alpha set at .028 in line with rough false discovery rate.

^b^Significant at the *P*<.028 level.

^c^Significant at the *P*<.001 level.

### What Is the Relationship Between Daily Problems, Coping, and Mood in Young People, as Measured by EMA Data?

As illustrated in Table 4, average happiness scores were negatively correlated with average sadness, anger, stress, and worry scores, and all negative mood scores were positively correlated with each other. Experience of daily problems was negatively correlated with happiness and significantly positively correlated with negative emotions of sadness, anger, stress, and worry. In contrast, higher coping efficacy was positively correlated with happiness and negatively correlated with sadness, anger, stress, and worry.

**Table 4:** Correlations between average mood, problems, and coping efficacy.

Variable^a^	1	2	3	4	5	6
1. Happy						
2. Sad	–.56					
3. Angry	–.45	.68				
4. Stressed	.42	.63	.48			
5. Worried	.40	.70	.52	.81		
6. Problems	.45	.45	.44	.60	.59	
7. Coping	.56	.56	.48	.56	.55	.70

^a^Alpha set at .026 in line with the rough false discovery rate. All correlations were significant at the *P*<.001 level.

To further explore these associations, a series of hierarchical linear regression were run (presented in Table 5), examining whether daily problems and coping efficacy predicted each of the 5 average mood states. Experience of problems was entered at Step 1, and coping efficacy was added at Step 2. Daily problems significantly predicted all mood states at Step 1, and for all analyses, the percentage variance explained increased from Step 1 to Step 2. At Step 2, daily problems became an insignificant predictor of happiness, sadness, and anger when coping efficacy was added to the model. In contrast, daily problems remained a significant predictor of stress and worry when coping efficacy was added. Coping efficacy significantly predicted all 5 mood states at Step 2.

**Table 5:** Hierarchical regressions predicting average mood.

Outcome		Adjusted *r*^2^	Predictor	Unstandardized coefficient, B	Standard error	β	*P* ^a^
**Happy**						
	Step 1	.19	Problems	–1.84	0.30	–.45^b^	<.001
	Step 2	.31	Problems	–0.43	0.40	–.10	.28
			Coping efficacy	1.41	0.28	.49^b^	<.001
**Sad**						
	Step 1	.20	Problems	2.20	0.36	.45^b^	<.001
	Step 2	.31	Problems	0.56	0.47	.17	.23
			Coping efficacy	–1.63	0.33	–.48^b^	<.001
**Angry**							
	Step 1	.19	Problems	1.96	0.33	.43^b^	<.001
	Step 2	.24	Problems	0.86	0.45	.19	.06
			Coping efficacy	–1.09	0.32	–.35^c^	.001
**Stressed**							
	Step 1	.36	Problems	3.32	0.36	.60^b^	<.001
	Step 2	.39	Problems	2.30	0.50	.42^b^	<.001
			Coping efficacy	–1.03	0.35	–.27^c^	. 004
**Worried**						
	Step 1	.35	Problems	3.08	0.34	.59^b^	<.001
	Step 2	.38	Problems	2.14	0.47	. 41^b^	<.001
			Coping efficacy	–0.94	0.33	–.26^c^	.005

^a^Alpha set at .028 in line with rough false discovery rate.

^b^Significant at the *P*<.001 level.

^c^Significant at the *P*<.028 level.

## Discussion

### Principal Findings

The aim of this study was to explore the use of a mobile phone app as a means of collecting EMA data pertaining to adolescent mood, problems, and coping in a school-based sample of young people. The researchers were interested in answering 4 broad questions, each of which is discussed below.

### To What Extent Do Participants Engage With the EMA Component of the App?

Engagement with the EMA component of the app was low compared with previous studies. For example, Kauer et al’s study reported that participants completed ratings on an average of 18 days during the 4-week intervention period [24], which was markedly higher compared with this study where participants completed ratings on an average of 5 days within the 28-day intervention period. However, in Kauer’s study, participants were given individualized summary reports of their data by their general practitioner at the end of the intervention period. This might have served as a motivational factor for adolescents to engage, either because they were interested in reviewing their data over time with a health professional or because they knew their general practitioner would be able to see their level of engagement and wanted to appear compliant. Similarly, Garcia et al reported an average response rate of 54% for EMA sampling using a similar methodology [23], compared with the average response rate of 18% obtained in this study, However, it is noteworthy that an incentive to engage was provided in Garcia’s study, whereby a higher level of engagement increased the number of entries they were given into a prize draw to win an iPod touch.

While participants were aware in this study that their EMA data would be recorded and accessible to the researchers, it was not emphasized that the main focus of the app was to collect information; the app was primarily presented to young people as an intervention as opposed to a data collection tool. It is possible that in studies where young people are aware of the importance of their data input toward achieving the research objective, engagement levels may be higher. For example, in Kauer et al’s study [24], participants were aware that their data were being collected, and that they would be able to review this with their general practitioner and receive a summary report on their data at the end of the study, which might have acted as an incentive for engagement. Thus, further research is necessary to establish response rate norms for adolescent populations using mobile phone EMA methodologies.

### Are EMA Responses Correlated With Standardized Measures of Mental Health and Coping?

Higher daily experience of problems was linked to more negative coping (as measured using a standardized instrument), suggesting that those who experience more problems or stressful events are more likely to engage in dysfunctional, avoidant coping strategies, in line with previous research [65-67]. However, a greater perceived ability to deal with daily problems was associated with higher levels of positive coping and lower levels of negative coping (as measured using a standardized instrument). This indicated that those who perceived themselves as dealing better with the problems they faced on a daily basis were using more positive coping strategies and less negative coping strategies, suggesting that this was a valid measure of coping self-efficacy. Similarly, as EMA mood ratings were correlated with standardized measures of distress and well-being, it suggested they had validity as indicators of adolescent mental health status.

### Are Brief Single-Item Measures of Key Protective Factors Related to Daily Mood States?

Findings indicated that the protective factors informal help-seeking, formal help-seeking, sleep, and sense of connectedness were associated with average daily mood states.

In terms of informal help-seeking, those who reported that they did not talk about their problems had more negative daily mood, which is consistent with previous research linking informal help-seeking and mental health [25,47-49]. Discussing problems may be considered a support-seeking coping strategy, which is likely to improve outcomes for young people by (1) providing them with instrumental help in addressing the source of their problems or in positively adapting to the situation, or (2) providing them with advice and support in taking steps toward achieving these goals themselves. To help promote talking about problems among adolescents, steps should be taken to ensure that school guidance counsellors have a high level of availability and that students know how to approach them confidentially in relation to problems they may be experiencing.

In terms of formal help-seeking, young people who identified themselves as having no problems unsurprisingly reported the least negative daily mood states. Interestingly, among those who reported that they had some problems, those who felt that they did not need help reported lower daily sadness than those who felt that they did need help, suggesting that young people have a good awareness of whether or not they need to seek help. This is in line with Rickwood et al’s [50] conceptualization of help-seeking as a process, which begins with the recognition that a problem exists for which help is required. Among those who reported a need to seek help, no significant difference emerged between those who had sought help and those who had not. Nonetheless, there was a trend toward lower stress and sadness and greater happiness among those who had sought help, indicating that seeking help is likely to be instigating some beneficial effects. However, there may be many uncontrolled factors at play, for example, severity of distress [68] or type of professional help obtained, that are known to affect outcomes of seeking professional help [69] which may explain why we do not see a statistically significant difference between those who have sought help and those who have not. These were beyond the scope of the analyses in this study but should be taken into account in future research.

Getting sufficient sleep was linked with more positive daily mood states, in line with literature in this area [58]. This indicates an important link between controllable health behavior and mood, and efforts should be made to promote sufficient sleep in young people. For example, parents can encourage appropriate bed times for adolescents on week-nights. At a policy level, education around sleep hygiene should be implemented into the SPHE (Social, Personal, and Health Education) curriculum for adolescents. Furthermore, consideration should be given to implementing later school start times in post-primary schools, which has been shown to be linked to a multitude of academic and well-being benefits for young people [70,71] by allowing them to get more sleep. Surprisingly, no correlation emerged between physical activity and daily mood states; however, the reason for this is unclear. There is evidence to suggest that adolescents may significantly overestimate their level of physical activity in research contexts [72], thus including some objective measure of physical activity (eg, mobile phone accelerometer data) would be useful in future research.

Finally, feeling a sense of connectedness and belonging was linked to more positive mental health outcomes for young people, in line with previous research [60-62]. Evidence suggests that this link between sense of belonging and mental health outcomes is likely to be bidirectional [60]. For example, rejection or exclusion from one’s social network means that one’s fundamental human need for connectedness is not met and is linked to increased symptoms of distress in adolescents [36,73]. However, young people who have mental health difficulties may experience cognitive distortions, which can cause them to view their interpersonal relationships in a more negative light [74,75], resulting in a diminished sense of belonging. Although sense of belonging is not as directly controllable as sleep and exercise, it can still be promoted in school, incorporating features that have been linked to increased sense of belonging among students, including appropriate policies and structures to prevent bullying, peer support programs, and extracurricular activities [76].

Overall, findings suggest that 4 of these brief items have good utility as indicators of the mental health status of an adolescent. This provides promise for the use of these items as a brief screening tool for young people. This may be useful as part of assessments in contexts where adolescents may not want to complete long measures of mental health outcomes, but where it would be useful for the clinician to have an overview of their mental health status, such as during general practitioner visits.

### What Is the Relationship Between Daily Problems, Coping, and Mood in Young People, as Measured by EMA Data?

Higher daily problems predicted lower happiness and higher negative affect, indicating that the more daily problems a young person experienced, the poorer their average daily mood was. This was consistent with previous research indicating that experience of negative daily events was associated with increased negative and decreased positive affect in adolescents [28,29]. In contrast, perceived coping efficacy was linked to greater happiness and lower negative affect, indicating that those who felt they had coped better with their problems experienced a more positive average daily mood.

However, coping efficacy did not moderate the relationship between problems and stress or worry. This suggested that even if young people were coping well, higher instances of daily problems were still associated with increased stress and worry. In contrast, coping efficacy did moderate the relationship between problems and happiness, sadness and anger, whereby when coping efficacy was taken into account, the relationship between problems and these mood states became negligible. Again, this was consistent with previous research indicating that coping effectively with problems was associated with more positive mood outcomes [30-32]. This is an important finding, as although we cannot prevent young people from experiencing problems in their lives, promoting the use of effective coping strategies among young people may help to offset the sadness and anger associated with the experience of such problems.

### Strengths and Limitations

To the authors’ knowledge this was the first study to explore the use of a mobile phone app as a method of EMA in a sample of Irish adolescents. A key strength of this study was the use of an electronic method of implementing EMA. This helped avoid problems associated with pen-and-paper EMA methods, such as falsifying the times at which EMA ratings were completed [10]; thus, increasing the ecological validity of the data captured.

In terms of limitations, the low level of app engagement was a significant issue. Interestingly, the level of engagement did not differ across key demographic variables, suggesting that the EMA analyses were unlikely to be biased in this regard. However, the low engagement levels observed pose a concern for future studies aiming to test hypotheses using adolescent EMA data collected via mobile phones. For example, unless a large number of participants are recruited, nonengagement and attrition over the course of the data collection period may result in an underpowered study. Furthermore, low levels of engagement over the course of study will result in high volumes of missing data, which pose a range of challenges to the data analyses [77,78]. Thus, despite its benefit in terms of ecological validity [1], caution must be exerted when considering the use of EMA with adolescents. In particular, advance considerations should be given to possible means of maximizing engagement such as personalized feedback from the app based on participants’ EMA responses [79] or, like in Kauer et al’s study, the provision of a summary report for participants at the end of the data collection period [24].

Future research should also give consideration to alternative methods of mobile phone EMA data collection. For example, advances in technology allow mobile phones to capture sensor data, such as Bentley et al’s Health Mashups app [80]. This app captured sensor data from users’ phones including their daily step count, sleep patterns, and GPS location in order to infer their patterns of health behaviors. It would be useful for future research to examine the validity and reliability of this type of sensor data, which does not require a high level of active engagement from participants in comparison to EMA self-report data.

Another potential issue in this study was that in the mood-rating section of the app, the sliding scale values were by default centered in the middle of the scale (at a value of 5). This meant it was possible that some users might have passively selected “Next” without moving the sliders to change the value, simply to complete the process of submitting an EMA rating rather than actively and accurately rating their mood. While it is unlikely that users navigated to this page unless they had the intention of completing a genuine mood rating, this still should be acknowledged as a potential design limitation of the app in this study.

It is also possible that the awareness that researchers were monitoring their app data might have resulted in participants using their phones differently to how they would if they were not being monitored, leading to response biases, such as socially desirable responding [81]. However, the researchers made an effort to emphasize the anonymous and confidential nature of the data to help avoid this.

Finally, it should be acknowledged that this was an exploratory study that employed simplistic analyses techniques. Thus, caution should be exerted in interpreting the generalizability of these findings. Future research is needed using more sophisticated analytical methods to explore EMA data captured over multiple time points, such as multilevel modeling, in order to robustly establish the validity of these findings in the population. The use of such techniques will also provide opportunities to model young people’s mood trajectories over time and test how these trajectories may be related to various demographic and well-being indicator variables.

### Conclusions

This study addressed a gap in the literature by exploring the validity and utility of mental health–related EMA data captured via mobile phone in a community sample of adolescents. Findings indicated a low level of engagement, suggesting that careful consideration must be given to ways of promoting participant engagement in order to maximize the amount of data obtained and ensure that the potential of these technologies is being sufficiently exploited. Despite the low engagement levels, analyses suggested that the data obtained were valid, correlating with standardized measures of coping, distress, and well-being. Analyses also revealed that EMA data can provide useful insights into the link between daily experience of problems, coping efficacy, and mood states in young people. These preliminary findings suggest that mobile phones have potential as valid and useful tools for EMA research in youth mental health. However, future research is required to robustly establish the validity of these findings among young people.

## Appendix

Multimedia Appendix 1 Number of participants who completed ecological momentary assessment (EMA) ratings on each day of the intervention period.

Multimedia Appendix 2 Average ecological momentary assessment (EMA) mood rating scores on each day of the intervention period.

## Abbreviations

ANOVA: analysis of variance
CSI: Coping Strategies Inventory
DASS-21: Depression, Anxiety, and Stress Scale
EMA: ecological momentary assessment
HOV: homogeneity of variance
WHO-5: World Health Organization Well-Being Index

## References

[1] Shiffman S, Stone AA, Hufford MR (2008). Ecological momentary assessment. Annu Rev Clin Psychol.

[2] Abbot BD, Uink B, Modecki K, Barber BL http://pandora.nla.gov.au/pan/141862/20160405-1343/www.youngandwellcrc.org.au/wp-content/uploads/2015/02/How-Do-You-Feel.pdf.

[3] Wenze SJ, Miller IW (2010). Use of ecological momentary assessment in mood disorders research. Clin Psychol Rev.

[4] Ebner-Priemer UW, Trull TJ (2009). Ecological momentary assessment of mood disorders and mood dysregulation. Psychol Assess.

[5] Tversky A, Kahneman D (1974). Judgment under uncertainty: heuristics and biases. Science.

[6] Koriat A, Goldsmith M, Pansky A (2000). Toward a psychology of memory accuracy. Annu Rev Psychol.

[7] Gorely T, Atkin AJ, Biddle SJ, Marshall SJ (2009). Family circumstance, sedentary behaviour and physical activity in adolescents living in England: Project STIL. Int J Behav Nutr Phys Act.

[8] Shrier LA, Shih MC, Beardslee WR (2005). Affect and sexual behavior in adolescents: a review of the literature and comparison of momentary sampling with diary and retrospective self-report methods of measurement. Pediatrics.

[9] Csikszentmihalyi M, Hunter J (2003). Happiness in everyday life: the uses of experience sampling. J Happiness Stud.

[10] Stone AA, Shiffman S, Schwartz JE, Broderick JE, Hufford MR (2003). Patient compliance with paper and electronic diaries. Control Clin Trials.

[11] Pew Research Center.

[12] Anderson M http://www.pewinternet.org/2015/10/29/technology-device-ownership-2015/.

[13] Lenhart A http://www.pewinternet.org/files/2015/04/PI_TeensandTech_Update2015_0409151.pdf.

[14] Harrison V, Proudfoot J, Wee PP, Parker G, Pavlovic DH, Manicavasagar V (2011). Mobile mental health: review of the emerging field and proof of concept study. J Ment Health.

[15] Torous J, Kiang MV, Lorme J, Onnela JP (2016). New tools for new research in psychiatry: a scalable and customizable platform to empower data driven smartphone research. JMIR Ment Health.

[16] Luxton DD, McCann RA, Bush NE, Mishkind MC, Reger GM (2011). mHealth for mental health: integrating smartphone technology in behavioral healthcare. Prof Psychol Res Pr.

[17] Mulvaney SA, Ho YX, Cala CM, Chen Q, Nian H, Patterson BL, Johnson KB (2013). Assessing adolescent asthma symptoms and adherence using mobile phones. J Med Internet Res.

[18] Piasecki TM, Trela CJ, Hedeker D, Mermelstein RJ (2014). Smoking antecedents: separating between- and within-person effects of tobacco dependence in a multiwave ecological momentary assessment investigation of adolescent smoking. Nicotine Tob Res.

[19] Grenard JL, Stacy AW, Shiffman S, Baraldi AN, MacKinnon DP, Lockhart G, Kisbu-Sakarya Y, Boyle S, Beleva Y, Koprowski C, Ames SL, Reynolds KD (2013). Sweetened drink and snacking cues in adolescents: a study using ecological momentary assessment. Appetite.

[20] Silk JS, Forbes EE, Whalen DJ, Jakubcak JL, Thompson WK, Ryan ND, Axelson DA, Birmaher B, Dahl RE (2011). Daily emotional dynamics in depressed youth: a cell phone ecological momentary assessment study. J Exp Child Psychol.

[21] Tan PZ, Forbes EE, Dahl RE, Ryan ND, Siegle GJ, Ladouceur CD, Silk JS (2012). Emotional reactivity and regulation in anxious and nonanxious youth: a cell-phone ecological momentary assessment study. J Child Psychol Psychiatry.

[22] Forbes EE, Stepp SD, Dahl RE, Ryan ND, Whalen D, Axelson DA, Birmaher B, Silk JS (2012). Real-world affect and social context as predictors of treatment response in child and adolescent depression and anxiety: an ecological momentary assessment study. J Child Adolesc Psychopharmacol.

[23] Garcia C, Hardeman RR, Kwon G, Lando-King E, Zhang L, Genis T, Brady SS, Kinder E (2014). Teenagers and texting: use of a youth ecological momentary assessment system in trajectory health research with latina adolescents. JMIR mHealth uHealth.

[24] Kauer SD, Reid SC, Crooke AH, Khor A, Hearps SJ, Jorm AF, Sanci L, Patton G (2012). Self-monitoring using mobile phones in the early stages of adolescent depression: randomized controlled trial. J Med Internet Res.

[25] Dooley B, Fitzgerald A http://www.ucd.ie/t4cms/MyWorldSurvey.pdf.

[26] Avenevoli S, Knight E, Kessler RC, Merikangas KR, Abela JRZ, Hankin BL (2008). The epidemiology of depression in children and adolescents. Handbook of Depression in Children and Adolescents.

[27] Kenny R, Dooley B, Fitzgerald A (2015). Feasibility of “CopeSmart”: a telemental heath app for adolescents. JMIR Ment Health.

[28] Schneiders J, Nicolson NA, Berkhof J, Feron FJ, van Os J, deVries MW (2006). Mood reactivity to daily negative events in early adolescence: relationship to risk for psychopathology. Dev Psychol.

[29] Larson R, Ham M (1993). Stress and “storm and stress” in early adolescence: the relationship of negative events with dysphoric affect. Dev Psychol.

[30] Ben-Zur H (2009). Coping styles and affect. Int J Stress Manag.

[31] Jaser SS, Champion JE, Dharamsi KR, Riesing MM, Compas BE (2011). Coping and positive affect in adolescents of mothers with and without a history of depression. J Child Fam Stud.

[32] Bordwine VC, Huebner ES (2010). The role of coping in mediating the relationship between positive affect and school satisfaction in adolescents. Child Ind Res.

[33] http://www.education.ie/en/Schools-Colleges/Services/DEIS-Delivering-Equality-of-Opportunity-in-Schools-/.

[34] Lovibond PF, Lovibond SH (1995). Manual for the Depression Anxiety Stress Scales. 2nd ed.

[35] Norton PJ (2007). Depression Anxiety and Stress Scales (DASS-21): psychometric analysis across four racial groups. Anxiety Stress Coping.

[36] Kenny R, Dooley B, Fitzgerald A (2013). Interpersonal relationships and emotional distress in adolescence. J Adolesc.

[37] Tully PJ, Zajac IT, Venning AJ (2009). The structure of anxiety and depression in a normative sample of younger and older Australian adolescents. J Abnorm Child Psychol.

[38] Bech P (2012). Clinical Psychometrics.

[39] Bech P, Gudex C, Johansen KS (1996). The WHO (Ten) well-being index: validation in diabetes. Psychother Psychosom.

[40] Bech P, Olsen LR, Kjoller M, Rasmussen NK (2003). Measuring well-being rather than the absence of distress symptoms: a comparison of the SF-36 Mental Health subscale and the WHO-Five well-being scale. Int J Methods Psychiatr Res.

[41] de Wit M, Pouwer F, Gemke RJ, Delemarre-van de Waal HA, Snoek FJ (2007). Validation of the WHO-5 well-being index in adolescents with type 1 diabetes. Diabetes Care.

[42] Möller Leimkühler AM, Heller J, Paulus NC (2007). Subjective well-being and 'male depression' in male adolescents. J Affect Disord.

[43] Aminzadeh K, Denny S, Utter J, Milfont TL, Ameratunga S, Teevale T, Clark T (2013). Neighbourhood social capital and adolescent self-reported wellbeing in New Zealand: a multilevel analysis. Soc Sci Med.

[44] Tobin DL, Holroyd KA, Reynolds RV, Wigal JK (1989). The hierarchical factor structure of the coping strategies inventory. Cogn Ther Res.

[45] Mullis AK, Mullis RL, Schwartz SJ, Pease JL, Shriner M (2007). Relations among parental divorce, identity status, and coping strategies of college age women. Identity.

[46] Perrone KM, Civiletto CL (2004). The impact of life role salience on life satisfaction. J Employ Couns.

[47] Piko BF, Fitzpatrick KM (2003). Depressive symptomatology among Hungarian youth: a risk and protective factors approach. Am J Orthopsychiatry.

[48] Schraedley PK, Gotlib IH, Hayward C (1999). Gender differences in correlates of depressive symptoms in adolescents. J Adolesc Health.

[49] Brown SL, Teufel JA, Birch DA, Kancherla V (2006). Gender, age, and behavior differences in early adolescent worry. J Sch Health.

[50] Rickwood D, Deane FP, Wilson CJ, Ciarrochi J (2005). Young people's help-seeking for mental health problems. Adv Mental Health.

[51] Sears HA (2004). Adolescents in rural communities seeking help: who reports problems and who sees professionals?. J Child Psychol Psychiatry.

[52] Saunders SM, Resnick MD, Hoberman HM, Blum RW (1994). Formal help-seeking behavior of adolescents identifying themselves as having mental health problems. J Am Acad Child Adolesc Psychiatry.

[53] Paluska SA, Schwenk TL (2000). Physical activity and mental health: current concepts. Sports Med.

[54] Biddle SJ, Asare M (2011). Physical activity and mental health in children and adolescents: a review of reviews. Br J Sports Med.

[55] Hallal PC, Victora CG, Azevedo MR, Wells JC (2006). Adolescent physical activity and health: a systematic review. Sports Med.

[56] World Health Organisation.

[57] Prochaska JJ, Sallis JF, Long B (2001). A physical activity screening measure for use with adolescents in primary care. Arch Pediatr Adolesc Med.

[58] Shochat T, Cohen-Zion M, Tzischinsky O (2014). Functional consequences of inadequate sleep in adolescents: a systematic review. Sleep Med Rev.

[59] Sleepfoundation.

[60] Jose PE, Ryan N, Pryor J (2012). Does social connectedness promote a greater sense of well-being in adolescence over time?. J Res Adolesc.

[61] Lee H, Williams RA (2013). Effects of parental alcoholism, sense of belonging, and resilience on depressive symptoms: a path model. Subst Use Misuse.

[62] Malaquias S, Crespo C, Francisco R (2014). How do adolescents benefit from family rituals? Links to social connectedness, depression and anxiety. J Child Fam Stud.

[63] Benjamini Y, Hochberg Y (1995). Controlling the false discovery rate: a practical and powerful approach to multiple testing. J R Stat Soc Series B Stat Methodol.

[64] West S, Finch J, Curran P, Hoyle R (1995). Structural equation models with nonnormal variables: problems and remedies. Structural equation modeling: Concepts, issues and applications.

[65] Rafnsson FD, Jonsson FH, Windle M (2006). Coping strategies, stressful life events, problem behaviors, and depressed affect. Anxiety Stress Coping.

[66] Windle M, Windle RC (1996). Coping strategies, drinking motives, and stressful life events among middle adolescents: associations with emotional and behavioral problems and with academic functioning. J Abnorm Psychol.

[67] Kraaij V, Garnefski N, de Wilde EJ, Dijkstra A, Gebhardt W, Maes S, ter Doest L (2003). Negative life events and depressive symptoms in late adolescence: bonding and cognitive coping as vulnerability factors?. J Youth Adolesc.

[68] Curry J, Rohde P, Simons A, Silva S, Vitiello B, Kratochvil C, Reinecke M, Feeny N, Wells K, Pathak S, Weller E, Rosenberg D, Kennard B, Robins M, Ginsburg G, March J (2006). Predictors and moderators of acute outcome in the Treatment for Adolescents with Depression Study (TADS). J Am Acad Child Adolesc Psychiatry.

[69] Kolko DJ, Brent DA, Baugher M, Bridge J, Birmaher B (2000). Cognitive and family therapies for adolescent depression: treatment specificity, mediation, and moderation. J Consult Clin Psychol.

[70] Boergers J, Gable CJ, Owens JA (2014). Later school start time is associated with improved sleep and daytime functioning in adolescents. J Dev Behav Pediatr.

[71] http://conservancy.umn.edu/bitstream/handle/11299/162769/Impact%20of%20Later%20Start%20Time%20Final%20Report.pdf?sequence=1&isAllowed=y.

[72] Sallis JF, Saelens BE (2000). Assessment of physical activity by self-report: status, limitations, and future directions. Res Q Exerc Sport.

[73] La Greca AM, Harrison HM (2005). Adolescent peer relations, friendships, and romantic relationships: do they predict social anxiety and depression?. J Clin Child Adolesc Psychol.

[74] Rose AJ, Carlson W, Luebbe AM, Schwartz-Mette RA, Smith RR, Swenson LP (2011). Predicting difficulties in youth's friendships: Are anxiety symptoms as damaging as depressive symptoms?. Merrill-Palmer Q.

[75] Prinstein MJ, Borelli JL, Cheah CS, Simon VA, Aikins JW (2005). Adolescent girls' interpersonal vulnerability to depressive symptoms: a longitudinal examination of reassurance-seeking and peer relationships. J Abnorm Psychol.

[76] Rowe F, Stewart D (2009). Promoting connectedness through whole‐school approaches: a qualitative study. Health Educ.

[77] Carpenter JR, Kenward MG http://citeseerx.ist.psu.edu/viewdoc/download?doi=10.1.1.468.9391&rep=rep1&type=pdf.

[78] Dziura JD, Post LA, Zhao Q, Fu Z, Peduzzi P (2013). Strategies for dealing with missing data in clinical trials: from design to analysis. Yale J Biol Med.

[79] Depp CA, Ceglowski J, Wang VC, Yaghouti F, Mausbach BT, Thompson WK, Granholm EL (2015). Augmenting psychoeducation with a mobile intervention for bipolar disorder: a randomized controlled trial. J Affect Disord.

[80] Bentley F, Tollmar K, Stephenson P, Levy L, Jones B, Robertson S, Price E, Catrambone R, Wilson J (2013). Health Mashups: presenting statistical patterns between wellbeing data and context in natural language to promote behavior change. ACM Trans Comput Hum Interact.

[81] Van de Mortel TF http://www.ajan.com.au/Vol25/Vol_25-4_vandeMortel.pdf.

