# Supplementary material for: Ecological Momentary Assessment of Adolescent Problems, Coping Efficacy, and Mood States Using a Mobile Phone App: An Exploratory Study
Source: JMIR Ment Health. 2016 Nov 29;3(4):e51. doi: 10.2196/mental.6361 (PMC5155083; doi:10.2196/mental.6361)

Multimedia Appendix 1: Number of participants who completed EMA ratings on each day of the intervention period.

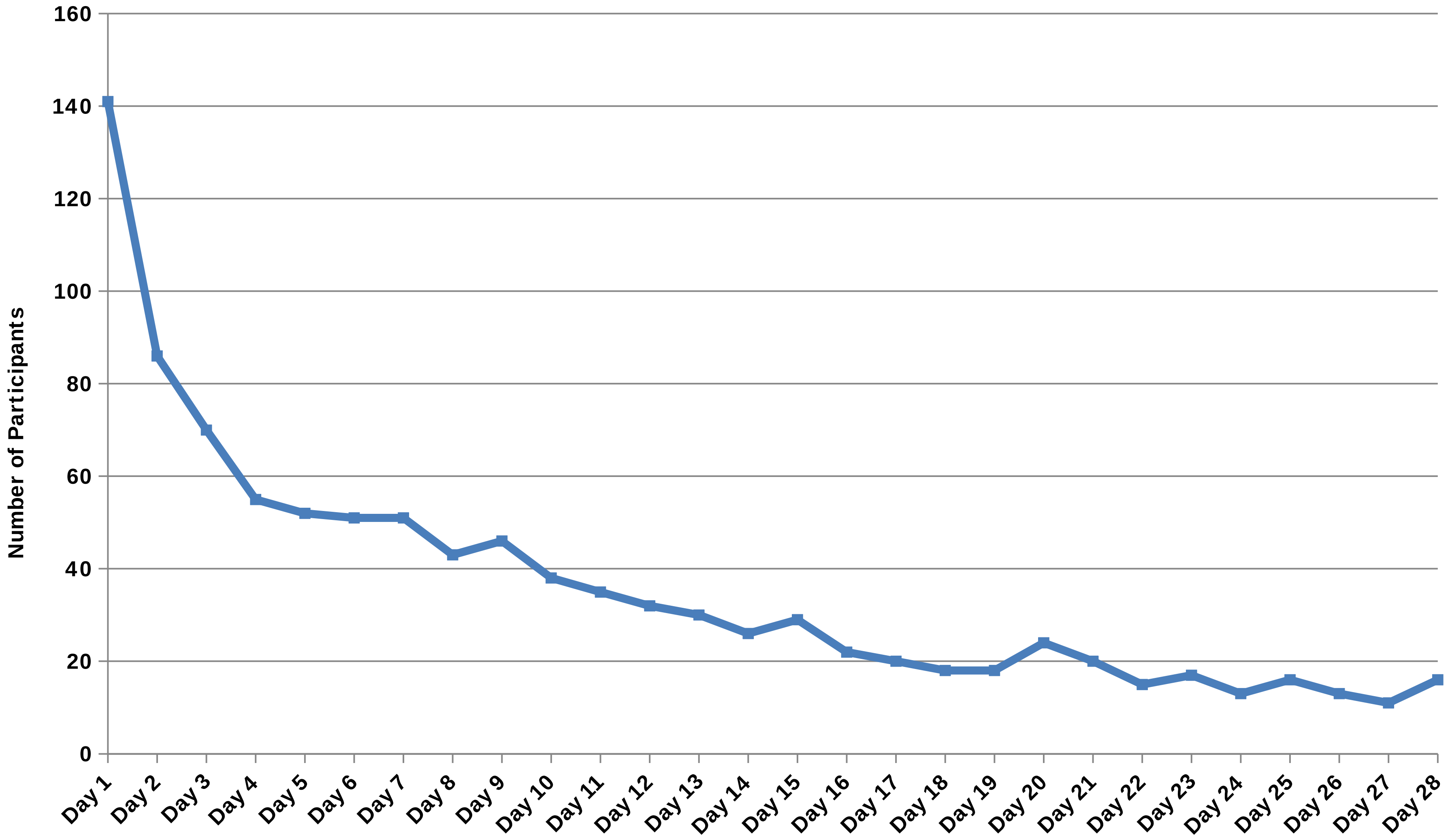

Supplement: Multimedia Appendix 1 [file mental_v3i4e51_app1.pdf]
