# Supplementary figures and images for: Ecological Momentary Assessment of Adolescent Problems, Coping Efficacy, and Mood States Using a Mobile Phone App: An Exploratory Study
Source: JMIR Ment Health. 2016 Nov 29;3(4):e51. doi: 10.2196/mental.6361 (PMC5155083; doi:10.2196/mental.6361)

Multimedia Appendix 2: Average EMA mood rating scores on each day of the intervention period.

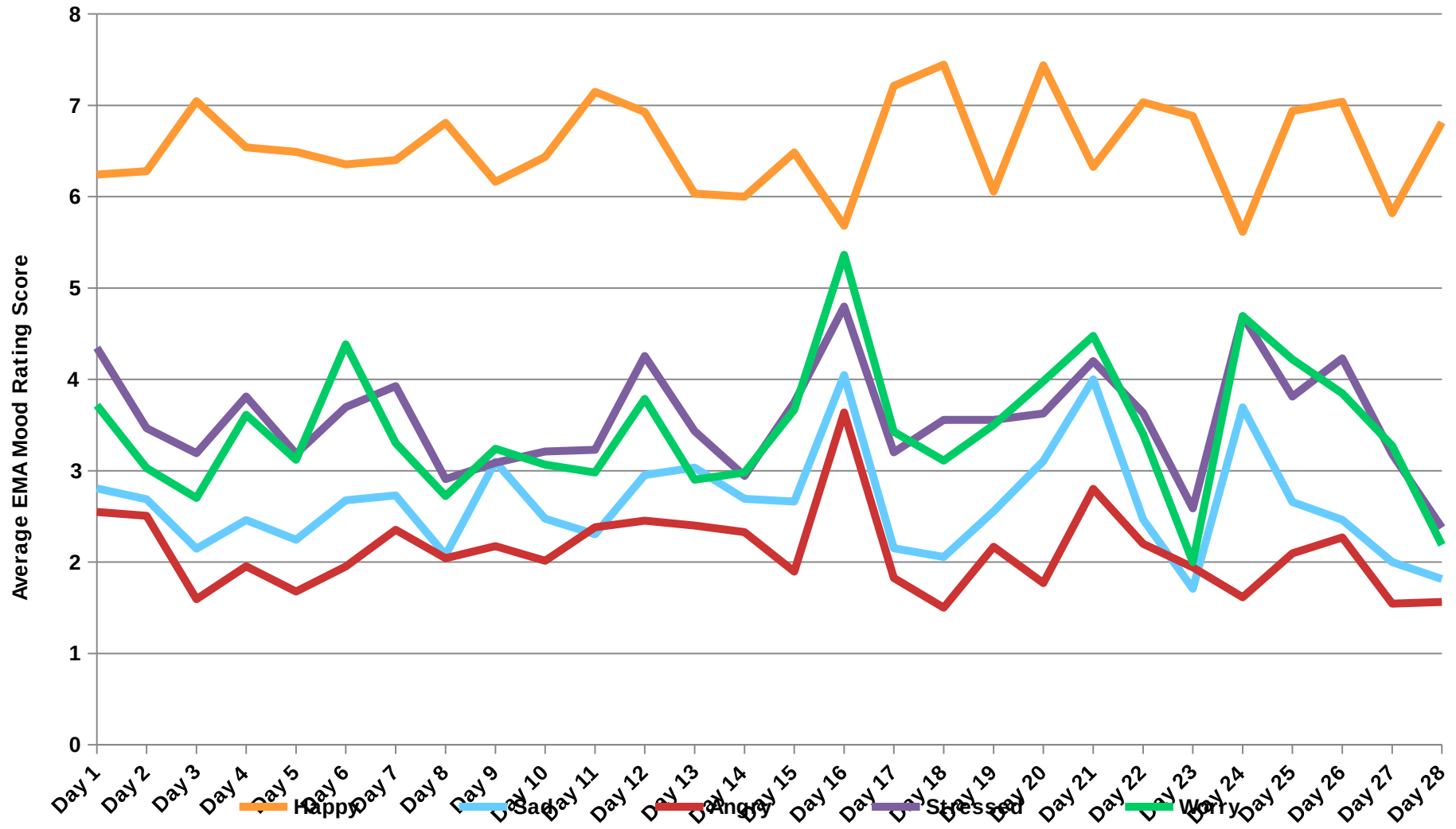

Supplement: Multimedia Appendix 2 [file mental_v3i4e51_app2.pdf]
